# Seven versus fourteen days for resistant bloodstream infections: what do we actually know? A scoping review

**DOI:** 10.1007/s15010-026-02821-y

**Published:** 2026-05-27

**Authors:** Valentina Zuccaro, Erika Asperges, Lea Nadia Marvulli, Pietro Valsecchi, Elisabetta Pagani, Paolo Sacchi, Raffaele Bruno

**Affiliations:** 1 Dipartimento di Scienze Cliniche Chirurgiche Diagnostiche e Pediatriche, Università di Pavia, 27100 Pavia, Italy; 2 SC Malattie Infettive I, Fondazione IRCCS Policlinico San Matteo, Pavia, Italy

**Keywords:** Antibiotic duration, Bloodstream infection, Antimicrobial resistance, ESBL, Carbapenemase, Scoping review

## Abstract

**Purpose:**

Recent randomized trials have established that 7 days of antibiotic therapy is noninferior to 14 days for bloodstream infections (BSI) caused by susceptible organisms. Whether this conclusion extends to BSI caused by resistant pathogens is unclear. We aimed to map the available evidence on antibiotic duration for resistant BSI, by resistance category, and to identify the gaps that must be addressed before shorter courses can be recommended in this setting.

**Methods:**

This scoping review was conducted following the Arksey and O’Malley framework and reported according to PRISMA-ScR guidelines. A search of PubMed was conducted through February 2026. We included RCTs, meta-analyses, and observational studies comparing antibiotic durations for BSI. Evidence was mapped across five resistance categories: ESBL-producing Enterobacterales, carbapenemase-producing Enterobacterales (CPE), MRSA, VRE, and MDR non-fermenting Gram-negatives.

**Results:**

We included 28 studies: 6 RCTs (5,182 patients, including the BALANCE pilot), 6 independent meta-analyses, 15 observational studies, and 1 post-hoc subgroup analysis of the BALANCE trial. All four definitive RCTs and all six meta-analyses confirmed noninferiority of 7-day therapy for susceptible BSI; none performed a resistance-stratified subgroup analysis for multidrug-resistant organisms (MROs). The only MRO-specific RCT (OPTIMISE, 2024) was halted after enrolling 103 of 382 planned patients. Observational data—including Le Berre et al. (379 ESBL-E BSI in ICU) (Le Berre et al. in Antibiotics (Basel) 14, 2025), Soto et al. (183 CRE BSI) (Soto et al. in Clin Infect Dis 78:27–30, 2024), Bahrs et al. (219 VRE BSI) (Bahrs et al. in Clin Microbiol Infect 29:200–207, 2023), and Gajdos et al. (EUROBACT-2, 550 hospital-acquired BSI) (Gajdos et al. in Intensive Care Med 51:518–528, 2025)—were consistent with no excess harm from shorter courses but were individually underpowered for noninferiority. For each of the five pre-specified resistance categories, evidence was either absent, insufficient for subgroup analysis, or derived from underpowered observational studies.

**Conclusion:**

Current RCT and meta-analytic evidence consistently supports the noninferiority of 7-day therapy for uncomplicated BSI caused by susceptible organisms. For resistant BSI, preliminary observational data are reassuring but far from definitive. Resistance-stratified trials are needed; the BALANCE + platform and SHORTEN-2 trial offer the most promising paths forward.

## Introduction

The traditional 10–14 day course of antibiotics for bloodstream infections (BSI) rested for decades on convention rather than evidence [1]. This has changed substantially. Between 2019 and 2025, four RCTs—Yahav et al. (604 patients) [2], von Dach et al. (503 patients, three-arm design) [3], Molina et al. (248 patients) [4], and the BALANCE trial (3,608 patients across 74 hospitals in 7 countries) [5]— each demonstrated noninferiority of 7-day versus 14-day therapy. Six independent meta-analyses, including a Bayesian analysis (97.8% probability of noninferiority) [6], a source-specific synthesis of urinary tract–sourced BSI (4,448 patients) [7], a trial sequential analysis confirming information size sufficiency [35], a meta-analysis incorporating both Gram-negative and Gram-positive BSI (4790 patients) [36], and the broadest synthesis to date including neonatal trials (5505 patients, 11 Randomized Controlled Trials—RCT-) [37], have confirmed these results. An individual participant data (IPD) meta-analysis of three pre-BALANCE RCTs (1186 patients) had previously shown consistent results across subgroups [38]. The question of duration for susceptible Gram-negative BSI can reasonably be considered settled.

What has not been settled is whether these findings extend to resistant organisms. Although three of the four definitive RCTs reported the prevalence of multi-drug resistant (MDR) organisms (Yahav: 18.0% MDR; von Dach: 5.0% extended-spectrum beta-lactamase (ESBL); Molina: 16.6% ESBL/AmpC), none performed resistance-stratified outcome analyses, with the exception of Yahav et al. who reported a subgroup analysis showing no difference in outcomes for MDR organisms (RD 0.1; 95% CI − 0.17 to 0.18) [2–4]. Methicillin-Resistant *Staphylococcus aureus* (MRSA) bacteremia was explicitly excluded. ESBL producers, carbapenemase-producing Enterobacterales (CPE), VRE, and MDR non-fermenters were either absent or present in unknown proportions. The single RCT designed specifically for MDR Gram-negative infections—the OPTIMISE trial [8]—was halted after enrolling only 103 of 382 planned patients, leaving the question unanswered.

A parallel body of observational literature has explored shorter courses in broader populations. Several of these studies included resistant organisms (14–17% ESBL producers in the cohorts of Sousa et al. [9], McAteer et al. [10], and Giannella et al. [11]), but none stratified duration outcomes by resistance. Two recent studies specifically examined resistant BSI: Le Berre et al. (379 ESBL-producing Enterobacteriaceae BSI in ICU) [12] and Soto et al. (183 CRE BSI) [13]. Neither found a significant difference, but neither had the power to establish noninferiority.

This scoping review was designed to map systematically, for the first time, what evidence exists—and what does not—on antibiotic duration for BSI across five distinct resistance categories. Our goal is not to synthesize effect estimates, which would be premature, but to define the landscape of the evidence gap and to identify where future research should be directed.

## Methods

### Study design and framework

We followed the five-stage framework of Arksey and O’Malley [14], as refined by Levac et al. [15], and reported according to the PRISMA-ScR checklist [16]. A scoping review was chosen over a systematic review because the objective was to map the extent of an evidence gap rather than to pool treatment effects—precisely the indication for which Munn et al. recommend this approach [17].

The research question, formulated using the Population–Concept–Context framework, was: “What evidence is available on optimal antibiotic duration (Concept) for BSI caused by antimicrobial-resistant organisms (Population) in hospital settings (Context)?” No protocol was prospectively registered in a public registry before the search. PROSPERO does not accept scoping review protocols, and at the time the review was conducted no equivalent mandatory registry existed for this study design. The research question, eligibility criteria, search string, and evidence-charting categories were pre-specified in an internal protocol finalized on 15 January 2026—i.e., before the PubMed search was executed on 15 February 2026—and were not modified during the review process. This protocol has subsequently been deposited in Zenodo for full transparency and is publicly accessible at https://doi.org/10.5281/zenodo.19641610. Readers who require direct access can also obtain it from the corresponding author upon request.

### Literature search

PubMed was searched on February 15, 2026 using the following string:

(“bloodstream infection” OR “bacteremia” OR “bacteraemia”) AND (“antibiotic duration” OR “duration of therapy” OR “treatment duration” OR “short course” OR “7 days” OR “14 days” OR “shorter”) AND (“gram-negative” OR “Enterobacteriaceae” OR “Enterobacterales” OR “resistance” OR “ESBL” OR “carbapenemase” OR “MRSA” OR “VRE” OR “multidrug-resistant”).

**Fig. 1:**
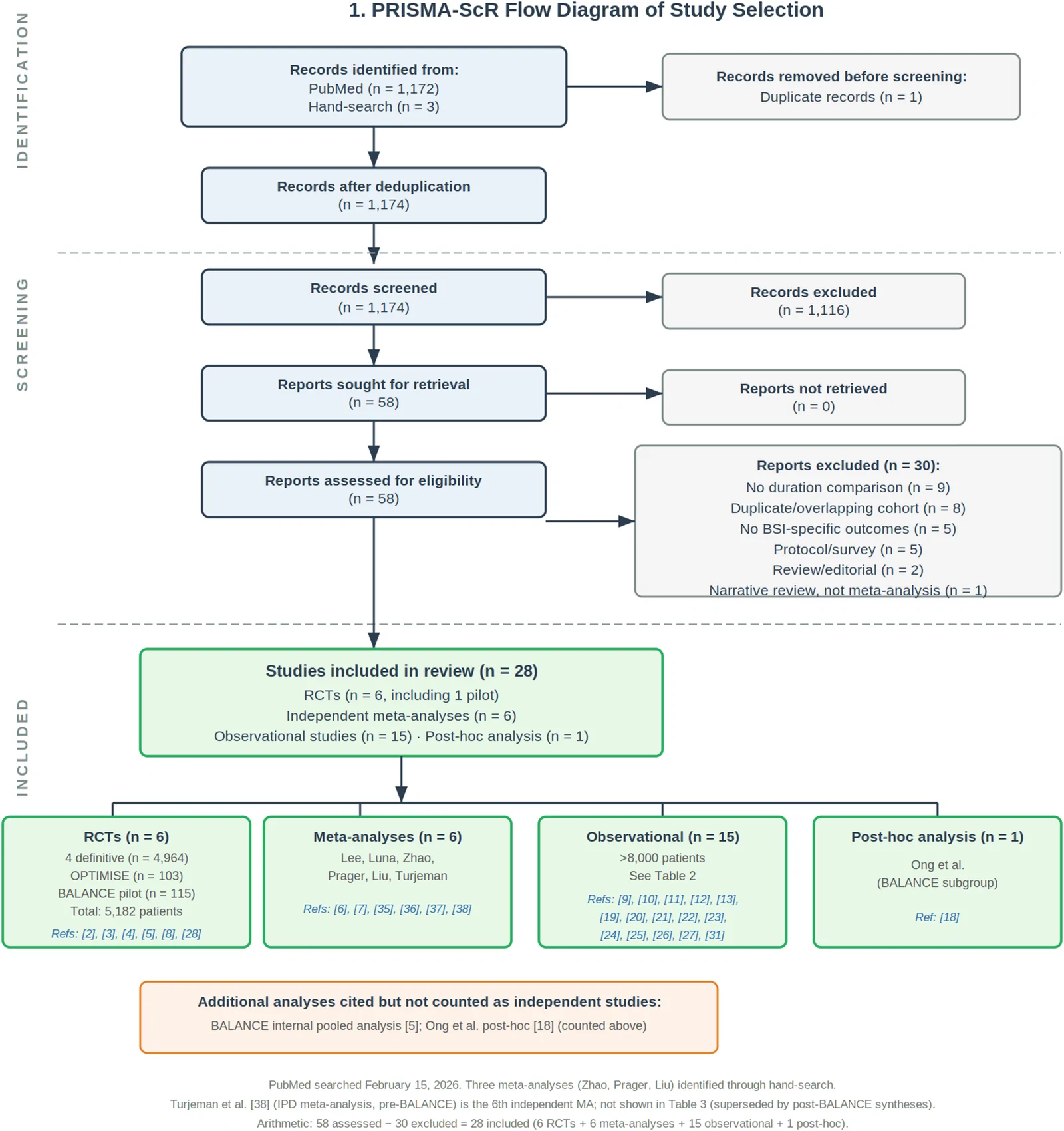
PRISMA-ScR flow diagram of study selection. PubMed searched on February 15, 2026, supplemented by hand-search of reference lists. Three meta-analyses (Zhao et al., Prager et al., Liu et al.) were identified through hand-search. The BALANCE internal pooled analysis [5] is cited in the text as part of the primary trial and is not counted separately; the post-hoc subgroup analysis by Ong et al. [18] is counted as a separate post-hoc analysis (n = 1) in the total of 28 included studies, distinct from the six independent meta-analyses

Reference lists of included studies, meta-analyses, and relevant guidelines were hand-searched. Only English-language publications were included. A single database was used, consistent with the mapping objective of this scoping review. The study selection process is depicted in Fig. 1.

### Eligibility criteria

We included: (1) RCTs comparing antibiotic durations for BSI in adults; (2) meta-analyses of such RCTs; (3) observational studies reporting duration-related outcomes for Gram-negative or Gram-positive BSI using methods to adjust for confounding. We excluded pediatric-only studies, studies of antibiotic choice rather than duration, and studies of non-BSI infections without a bacteremia subgroup. Two independent reviewers screened the titles, abstracts, and full texts.

### Data charting

We extracted study design, sample size, setting, pathogen distribution, resistance data, duration definitions, outcomes, and statistical methods. Evidence was mapped across five pre-specified resistance categories: ESBL-producing Enterobacterales, CPE, MRSA, VRE, and MDR non-fermenting Gram-negatives (including difficult-to-treat *P. aeruginosa*, carbapenem-resistant *A. baumannii*, and *S. maltophilia*). No risk-of-bias assessment or meta-analytic synthesis was performed, consistent with scoping review methodology [14, 15, 17].

## Results

### Overview

The search and selection process yielded 28 studies for inclusion (Fig. 1): 6 RCTs totalling 5,182 patients (4 definitive trials with 4,964 patients [2–5], OPTIMISE with 103 patients [8], and the BALANCE pilot with 115 patients [28]), 6 independent meta-analyses [6, 7, 35–38], 15 observational studies [9–13, 19–27, 31], and 1 post-hoc subgroup analysis of the BALANCE trial [18]. Tables 1, 2, and 3 summarize the RCTs, observational studies, and meta-analyses, respectively.

**Table 1:** RCTs on antibiotic duration for BSI

Trial	N	Setting	Pathogens	Resistance Data	Outcome (shorter treatment)	Refs.
BALANCE (2025)	3,608	74 sites, 7 countries	GN ~ 71%, GP ~ 17%, polymicrobial ~ 12%	S. aureus excluded; resistance not reported	90—d mortality: noninferior	[5]
Yahav (2019)	604	3 sites (Israel, Italy)	GN 100%	MDR: 109/604 (18.0%)	Composite at 90—d: noninferior	[2]
von Dach (2020)*	503	3 sites (Switzerland)	Enterobacterales 100%	ESBL: 40/503 (7.95%)	30—d clinical failure: noninferior	[3]
Molina (2022)	248	5 sites (Spain)	Enterobacterales 100%	ESBL: 28/237 (11.3%) AmpC: 13/247 (5,3%)	Reduction of total treatment days with noninferiority of secondary outcomes	[4]
OPTIMISE (2024)	103	18 ICUs, Brazil	MDR-GNB 100%	100% MDR	28—d failure: noninferiority not demonstrated (early termination)	[8]
BALANCE pilot (2018)	115	11 ICUs, Canada	GN and GP	Not reported	90—d mortality: no analyses per treatment group	[28]

*GN*, Gram-negative; *GP*, Gram-positive; *MDR-GNB*, multidrug-resistant Gram-negative bacteria. *Three-arm trial. Resistance phenotype definitions vary across trials: Yahav et al. applied the following definition for MDR: Enterobacteriaceae demonstrating resistance to oxyimino-beta-lactam substrates (cefotaxime and ceftazidime), Pseudomonas spp. resistant to three or more of the following antimicrobial agents: antipseudomonal penicillins (e.g., piperacillin), antipseudomonal cephalosporins (e.g., ceftazidime), fluoroquinolones (e.g., ciprofloxacin), carbapenems (imipenem, meropenem), and aminoglycosides, Acinetobacter spp. resistant to three or more of the following agents: imipenem (or meropenem), levofloxacin (or other fluoroquinolones), ceftazidime, colistin, tobramycin (or other aminoglycosides), and piperacillin–tazobactam; von Dach et al. reported ESBL producers only; Molina et al. combined ESBL and AmpC producers; BALANCE did not report resistance status; OPTIMISE enrolled exclusively MDR Gram-negative isolates and reports ESBL, KPC, and MDR P. aeruginosa subgroups separately

**Table 2:** Observational studies on antibiotic duration for BSI

Study	N	Design	Short	Long	Outcome	Resistance	Key finding
Chotiprasitsakul (2018) [20]	770	Retrospective, PS-matched, multicenter	6–10 d	11–16 d	30—d mortality	Not stratified	No difference, HR 1.12 (0.70–1.80); trend toward less MDRGN emergence
Giannella (2018) [11]	856	Retrospective, single-center	≤ 10 d	> 10 d	90—d mortality	*E. coli* (not stratified)	No excess mortality or relapse
Nelson (2017) [19]	411	Retrospective, PS-adjusted	7–10 d	> 10 d	90—d failure	Not reported	13.2% (short) vs 8.5% (long)
Sousa (2019) [9]	395	Prospective, PS-stratified	7–10 d	> 10 d	30—d mortality	14% ESBL, 4% CPE	No difference in general population
McAteer (2023) [10]	1,099	Retrospective cohort, multicenter	7 or 10 d	14 d	30—d recurrence	13% ESBL, 1% CPE	aOR 0.99 (95% CI 0.52–1.87)
Tingsgård (2024) [21]	1,040	Target trial emulation, multicenter	5–7 d	> 7 d	90—d mortality	0.4% ESBL	No difference
Tingsgård (2024) [22]	914	Target trial emulation, multicenter	IV → oral ≤ 4 d	IV ≥ 5 d	90—d mortality	10.9% ciprofloxacin-resistant	Early switch comparable (RR 0.78; 0.60–1.10)
Zuercher (2023) [23]	382	Retrospective cohort, multicenter	≤ 10 d	> 10 d	Time to discharge/death	Mixed (not stratified)	No benefit of longer therapy
Le Berre (2025) [12]	379	Retrospective cohort, multicenter	≤ 7 d	> 7 d	Mortality	100% ESBL-E	HR 1.65 (0.91–3.00)
Soto (2024) [13]	183	Retrospective, PS-weighted, multicenter	7–10 d	14–21 d	30—d composite	100% CRE	OR 1.21 (0.55–2.31)
Bahrs (2023) [25]	219	Retrospective, multicenter, IPTW	≤ 9 d	≥ 10 d	30—d mortality	100% VRE	OR 1.15 (p = 0.773); shorter hospital stay
Abbas (2020) [26]	530	Retrospective cohort, single-center	≤ 14 d	> 14 d	90—d mortality	17.7% MRSA	aHR 0.32 for > 14 d in complicated SAB; aHR 0.85 (0.41–1.78) uncomplicated SAB
Evans (2023) [27]	587	Retrospective, cloning approach	≤ 10 d	> 10 d	28—d in-hospital mortality	15.7% MRSA	HR 1.74 (1.36–2.24) for short therapy; no significant interaction by methicillin susceptibility (P = 0.12)
Torres (2025) [31]	48	Retrospective, single-center	≤ 6 d	> 6 d	30—d recurrence	100% VRE	No recurrences; mortality 30% vs 21.1% (p = 0.41)
Gajdos (2025) [24]	550	Prospective cohort, multicenter, IPTW	7–10 d	14–21 d	28—d treatment failure	17% DTR-GN (short); 36.6% DTR-GN (long)	OR 0.64 (0.44–0.93)

*PS*, propensity score; *d*, days; *DTR-GN*, difficult-to-treat Gram-negative; *SAB*, S. aureus bacteremia; *IPTW*, inverse probability of treatment weighting

**Table 3:** Meta-analyses of antibiotic duration for BSI

Meta-analysis	Studies	Patients	Population	Primary Outcome	Conclusion
Lee et al. (2025) [6]	4 RCTs	4,795	GN BSI, adults	90—d mortality: RR 0.93 (0.81–1.07); Bayesian: 97.8% probability of noninferiority	7—d noninferior to 14—d (frequentist and Bayesian)
Luna et al. (2026) [7]	3 RCTs + 3 obs	4,448	GN BSI from UTI source	90—d mortality: RR 0.91 (0.72–1.15); 30—d mortality: RR 0.97 (0.64–1.47)	7—d noninferior for UTI-sourced BSI
Zhao et al. (2025) [35]	4 RCTs	4,794	Uncomplicated BSI, adults	All-cause mortality: RR 0.96 (0.73–1.25); 90—d mortality: RR 0.94 (0.80–1.10); TSA performed	No difference; TSA confirms information size sufficiency
Prager et al. (2025) [36]	4 RCTs	4,790	BSI (GN and GP), adults	90—d mortality: RR 0.93 (0.81–1.07); prediction interval 0.74–1.17	Similar efficacy and safety; includes GP subgroup
Liu et al. (2025) [37]	11 RCTs	5,505	Immunocompetent BSI (neonates and adults)	Mortality: RR 0.91 (0.79–1.05); LOS: MD − 3.04 d (− 3.9 to − 2.18)	No difference in mortality; shorter LOS with short course

*GN*, Gram-negative; *GP*, Gram-positive; *BSI*, bloodstream infection; *UTI*, urinary tract infection; *TSA*, trial sequential analysis; *LOS*, length of hospital stay. Turjeman et al. [38] (IPD meta-analysis of 1,186 patients from three pre-BALANCE RCTs) is not shown because it has been superseded by the larger post-BALANCE meta-analyses. The BALANCE internal pooled analysis [5] is cited in the text as part of the primary trial [5], and Ong et al. [18] is reported separately as a post-hoc subgroup analysis of BALANCE (counted under ‘Post-hoc analysis (n = 1)’ in Fig. 1); neither meets the definition of an independent meta-analysis

### Randomized Controlled trials

Six randomized controlled trials informing the question of antibiotic duration for BSI have now been published (Table 1). Four of these—Yahav et al. (2019, n = 604) [2], von Dach et al. (2020, n = 503) [3], Molina et al. (2022, n = 248) [4], and the BALANCE trial (2025, n = 3,608) [5]—are definitive phase-III noninferiority trials and collectively enrolled 4,964 patients with predominantly Gram-negative BSI from community and hospital-acquired sources. All four demonstrated noninferiority of 7-day versus 14-day therapy for their respective primary endpoints (90-day mortality or composite clinical failure), and none reported a safety signal favouring the longer-course arm.

Three of the four definitive RCTs reported the prevalence of multidrug-resistant (MDR) organisms—Yahav et al.: 18.% MDR [2]; von Dach et al.: 8.0% ESBL producers [3]; Molina et al.: 16.6% ESBL/AmpC producers [4]—but only Yahav et al. performed a pre-specified resistance-stratified subgroup analysis, which showed no difference in the primary composite outcome for patients with MDR isolates (risk difference 0.1; 95% CI − 0.17 to 0.18) [2]. The BALANCE trial excluded *S. aureus* bacteremia and did not report the proportion of resistant Gram-negative isolates [5]. Carbapenemase-producing Enterobacterales (CPE), vancomycin-resistant enterococci (VRE), and MDR non-fermenters were either absent or present in unknown proportions across all four definitive trials.

The BALANCE pilot (n = 115) [28] established feasibility but was not powered for comparative outcomes. The OPTIMISE trial [8] is the only RCT specifically designed for MDR Gram-negative BSI, enrolling critically ill patients from 18 Brazilian ICUs; it was halted after recruiting 103 of the 382 planned patients (27% of target), and noninferiority was not demonstrated. The intention-to-treat 28-day clinical failure rates (42.4% vs 44.7%; 95% CI − 21.3 to 16.7) were numerically reassuring but uninformative given the width of the confidence interval, while the per-protocol analysis suggested an excess of new Gram-negative infections in the 7-day arm (23.4% vs 3.6%)—a finding we discuss further below.

### Observational evidence: resistance-related findings

The observational literature on BSI duration now includes 15 studies involving over 8,000 patients. While most were designed to compare shorter versus longer courses irrespective of resistance, several contain data relevant to the question of whether shorter therapy is safe for resistant organisms.

A consistent, though never statistically significant, finding across studies is a trend toward less emergence of resistant organisms with shorter antibiotic courses. Chotiprasitsakul et al. (2018), in a propensity-matched analysis of 770 patients across 3 US centers, reported a trend toward reduced MDRGN emergence with short-course therapy (OR 0.59; P = 0.09) [20]. McAteer et al. (2023), in the largest observational study to date (1,099 patients with UTI-associated bacteremia, 13% ESBL producers), found numerically fewer resistant recurrences with shorter therapy, although the difference was not significant (aOR 0.99; 95% CI 0.52–1.87) [10]. While no individual study was powered for this outcome, the consistency of direction across independent datasets lends biological plausibility.

Gajdos et al. (2025), using Inverse Probability of Treatment Weighting (IPTW) analysis from the international EUROBACT-2 cohort (550 ICU patients with hospital-acquired BSI), found that short-course treatment (7–10 days) was associated with reduced 28-day treatment failure compared with prolonged treatment (14–21 days; OR 0.64; 95% CI 0.44–0.93) [24]. Notably, difficult-to-treat Gram-negative organisms were present in 17% of the short-course group and 36.6% of the long-course group.

Two methodological themes from the observational literature are particularly relevant to resistant BSI. First, the role of antibiotic bioavailability: Nelson et al. (2017) reported higher treatment failure with shorter courses overall (HR 2.60; 95% CI 1.20–5.53) [19], but this excess risk disappeared when restricting the analysis to patients receiving intravenous (IV) or highly bioavailable oral agents (HR 0.33). McAteer et al. confirmed this finding (aOR 0.76 for bioavailable agents vs aOR 2.54 overall) [10]. Second, the methodological challenge of immortal time bias has been addressed through target trial emulation (Tingsgård et al. [21, 22]) and cloning approaches (Zuercher et al. [23], Evans et al. [27]).

### Evidence map by resistance category

For each of the five pre-specified resistance categories, we summarize the evidence hierarchically, presenting randomized data (definitive RCTs, pilot trials, and stopped trials) first, followed by observational data (prospective and retrospective cohort studies, target trial emulations, and propensity-weighted analyses). The design of every cited study is stated explicitly to prevent unintended equivalence between randomized and non-randomized evidence.

### ESBL-producing Enterobacterales

Terminology for β-lactamase-mediated resistance is not used uniformly across the primary studies: Molina et al. [4] reports ESBL and AmpC producers combined (16.6%), Yahav et al. [2] reports a composite MDR category (17.9%) that includes both ESBL and carbapenem-resistant isolates, and von Dach et al. [3] reports ESBL producers only (8.0%); Le Berre et al. [12] is restricted to ESBL-producing Enterobacteriaceae by design. Where the source study combines phenotypes, we use the term ‘ESBL/AmpC’; otherwise, we preserve the original designation used by the authors.

#### Randomized evidence

Three of the four definitive RCTs reported MDR prevalence: Yahav et al. [2] (RCT; 18.0% MDR; resistance-stratified risk difference 0.1, 95% CI − 0.17 to 0.18), von Dach et al. [3] (RCT; 8.0% ESBL), and Molina et al. [4] (RCT; 16.6% ESBL/AmpC). The BALANCE trial [5] did not report resistance status. OPTIMISE [8] (stopped RCT; 12.2% ESBL among n = 103) is too small for subgroup inference.

#### Observational evidence

Le Berre et al. [12]—a retrospective multicentre cohort of 379 ICU patients with ESBL-producing Enterobacteriaceae BSI—found no significant mortality difference between ≤ 7 and > 7 days (HR 1.65; 95% CI 0.91–3.00), though the point estimate favoured longer therapy. Five additional observational studies [9–11, 20, 24] included ESBL isolates at prevalences of 14–17% but did not stratify outcomes by resistance.

### Carbapenemase-producing enterobacterales

#### Randomized evidence

No definitive RCT enrolled or stratified CPE patients. OPTIMISE [8] (stopped RCT) included 10.8% KPC producers, but the numbers were too small for CPE-specific conclusions.

#### Observational evidence

Soto et al. [13]—a retrospective propensity-weighted multicentre cohort of 183 CRE BSI patients across 24 US hospitals—found no difference in the 30-day composite outcome between 7–10 and 14–21 days of therapy (OR 1.21; 95% CI 0.55–2.31). The IDSA AMR Guidance (2024) offers no duration recommendations for CPE BSI [29].

### MRSA

#### Randomized evidence

*S. aureus* bacteremia was explicitly excluded from all four definitive RCTs and from the BALANCE pilot. The SAB7 RCT (NCT03596983) [30] is ongoing.

#### Observational evidence

Abbas et al. [26] (retrospective single-centre cohort, n = 530, 17.7% MRSA) reported that > 14 days of therapy was associated with improved survival in complicated SAB (aHR 0.32; 95% CI 0.16–0.64) but not in uncomplicated SAB (aHR 0.85; 0.41–1.78). Evans et al. [27] (retrospective cohort with a cloning approach to address immortal-time bias, n = 587, 15.7% MRSA) reported higher 28-day mortality for < 10 days of therapy compared with > 18 days (HR 1.74; 95% CI 1.36–2.24), with no significant interaction by methicillin susceptibility (P = 0.12).

### VRE

#### Randomized evidence

The BALANCE trial [5] enrolled ~ 6.9% enterococcal BSI, but vancomycin susceptibility was not reported. No RCT has specifically addressed VRE BSI duration.

#### Observational evidence

Bahrs et al. [25] (IPTW-adjusted retrospective multicentre cohort, 219 patients with uncomplicated VRE bacteremia) found no difference in 30-day mortality (19.2% vs 22.0%; adjusted OR 1.15; p = 0.773), with shorter hospital stays in the short-course group. Torres et al. [31] (retrospective single-centre cohort, 48 patients with uncomplicated VRE bacteremia) found no bacteremia recurrence in either group, with comparable mortality (30-day: 30% vs 21.1%, p = 0.41).

### MDR non-fermenting gram-negatives

#### Randomized evidence

*Pseudomonas* species accounted for 4.7% of BALANCE [5] isolates (not stratified by resistance). OPTIMISE [8] (stopped RCT) enrolled MDR P*. aeruginosa* (6.8%) and *Acinetobacter* spp.; the per-protocol analysis raised a concern, with new GNB infections occurring in 23.4% of the 7-day group versus 3.6% of the 14-day group. The SHORTEN-2 RCT (NCT05210439) [32], designed specifically for *P. aeruginosa* BSI across 30 Spanish hospitals, is currently recruiting.

#### Observational evidence

No dedicated observational study for MDR non-fermenting Gram-negatives has been published to date; relevant data are embedded in mixed-cohort analyses (e.g., Gajdos et al. [24] reported 17% DTR-GN in the short-course group and 36.6% in the long-course group) but are not stratified by resistance phenotype.

### Meta-analyses

Six independent meta-analyses of randomized evidence on antibiotic duration for BSI were retrieved (Table 3). Lee et al. (2025) [6] performed both a frequentist and a Bayesian synthesis of the four definitive RCTs (4,795 patients) and reported a posterior probability of noninferiority of 97.8%. Luna et al. (2026) [7] synthesized evidence restricted to urinary-tract-sourced BSI (4,448 patients). Zhao et al. (2025) [35] added a trial sequential analysis demonstrating that the information size for mortality had been reached. Prager et al. (2025) [36] extended the synthesis to include both Gram-negative and Gram-positive BSI (4,790 patients). Liu et al. (2025) [37] provided the broadest synthesis to date (11 RCTs, 5,505 participants) by including neonatal trials. Turjeman et al. (2022) [38]—an IPD meta-analysis of three pre-BALANCE RCTs—preceded the BALANCE trial and has been effectively superseded by the post-BALANCE syntheses; it is cited in the text for completeness but not tabulated.

Crucially, none of these six meta-analyses performed a resistance-stratified subgroup analysis for MDR organisms. This is not a methodological oversight but a direct consequence of the underlying trials: because the definitive RCTs either excluded resistant isolates (e.g., MRSA, CPE), enrolled them in small and imprecisely reported proportions, or did not report resistance-stratified outcomes, the individual-study data required for a pooled MDR organism subgroup analysis simply do not exist. The meta-analytic evidence therefore confirms noninferiority of 7-day therapy in susceptible BSI with high precision but provides no direct information on resistant BSI. This absence is itself one of the principal findings of the present review and the main motivation for the evidence map presented in Table 4.

**Table 4:** Evidence map by resistance category

Category	RCT evidence	Observational	Ongoing RCTs	Key gap
ESBL-E	OPTIMISE: 12.2%(n = 103) Yahav: 18.05% (n = 109) Molina: 11.33% (n = 28) von Dach: 7.95% (n = 40)	5 cohorts include ESBL (14–17%); 1 ESBL-specific (n = 379)	None	No powered subgroup; resistance profiles unreported in definitive RCTs
CPE	OPTIMISE: 10.8% KPC (n = 103)	1 study (n = 183 CRE)	None	Near-complete exclusion from RCTs; IDSA 2024 offers no duration guidance
MRSA	Excluded from all RCTs	Evans: 15.7% (n = 92) Abbas: 17.7% (n = 94)	SAB7	No RCTs; no powered subgroup; need to account for complication status
VRE	Not reported (enterococcal ~ 6.9% in BALANCE; susceptibility unknown)	Bahrs: 100% (n = 219) Torres: 100% (n = 48)	None	No RCTs; only small retrospective data
MDR non-fermenters	OPTIMISE: MDR-PA 6.8% (n = 103); per-protocol signal (new GNB: 23.4% vs 3.6%)	None	SHORTEN-2	Underpowered; signal in per-protocol analysis warrants dedicated trial

*ESBL-E*, ESBL-producing Enterobacterales; *CPE*, carbapenemase-producing Enterobacterales; *MDR-PA*, multidrug-resistant *P. aeruginosa*

## Discussion

The central finding of this review is that there is a discrepancy between areas where evidence is strong and areas where it is needed. For susceptible Gram-negative BSI, the case for 7-day therapy rests on four RCTs, six independent meta-analyses, and a convergent body of observational data. For resistant BSI, where decisions about duration are both most consequential and most uncertain, we found either no data or data from underpowered studies. This is not because no one has tried. The OPTIMISE trial was explicitly designed to fill this gap, but it enrolled only 27% of its target sample before being halted for slow recruitment [8]. The ITT results (42.4% vs 44.7% clinical failure) were reassuring, but the confidence interval (− 21.3 to 16.7) was far too wide to conclude anything. More concerning was the per-protocol finding of higher new GNB infections in the 7-day group (23.4% vs 3.6%)—a result that warrants attention regardless of whether it reflects a true biological signal or the instability of a stopped trial.

A methodological point deserves emphasis. The absence of a statistically significant difference in underpowered studies—whether OPTIMISE, Le Berre, or Soto—should not be interpreted as evidence of equivalence. This distinction between absence of evidence and evidence of absence carries particular weight when applied to clinical decisions in populations with higher baseline mortality and fewer therapeutic alternatives [8, 12, 13].

The observational evidence for resistant BSI leans in a reassuring direction. Le Berre et al. found no significant mortality difference for ESBL-producer BSI in ICU, but the point estimate (HR 1.65) actually favored longer therapy and the confidence interval crossed 1 [12]. Soto et al. found no difference for CRE BSI (OR 1.21; 95% CI 0.55–2.31), but with 183 patients, this study could not have detected clinically important differences [13].

Two themes from the observational literature deserve attention in the context of resistant BSI. First, the role of bioavailability: if optimal duration depends on the pharmacokinetic properties of the antibiotic [10, 19], this has direct implications for resistant infections where highly bioavailable oral options are often unavailable. Second, the trend toward reduced resistance emergence with shorter courses [20, 23] is particularly relevant in a population already colonized or infected with resistant organisms.

The clinical question facing physicians today is practical: when treating ESBL-producing *E. coli* or KPC-producing *K. pneumoniae* BSI with an active agent and adequate source control, is 7 days sufficient? A biologically plausible argument can be made that duration should depend on the activity of the chosen antibiotic rather than the resistance mechanism per se. But patients with resistant BSI differ from those with susceptible BSI in ways that may matter independently: they tend to be older, more comorbid, more likely to have healthcare-associated infections, and more likely to have received prior antibiotics. The MERINO trial serves as a cautionary example of how in vitro activity can fail to predict clinical outcomes [33]. Importantly, while severely immunocompromised patients have been systematically excluded from the definitive RCTs, the BALANCE trial did enroll over 1,900 ICU patients and demonstrated noninferiority across prespecified subgroups. The gap in evidence therefore applies specifically to patients with severe immunosuppression rather than to critically ill patients more broadly.

### Future research

Standalone noninferiority trials for any single resistance category would require approximately 4,000 patients per arm. The most realistic path forward is the BALANCE + adaptive platform trial [34], which could incorporate resistance-stratified duration domains into its existing multi-center infrastructure. The SHORTEN-2 trial [32] may contribute specific evidence for *P. aeruginosa* BSI. In the interim, pooling individual patient data from the four definitive RCTs with resistance-level microbiological data could provide preliminary resistance-stratified estimates.

### Limitations

Several limitations should be noted. We searched PubMed only, supplemented by hand-search of reference lists; three meta-analyses were identified through hand-search rather than the primary database search. Studies indexed exclusively in Embase, CENTRAL, or regional databases may have been missed. No protocol was prospectively registered in a public registry before searching, which we acknowledge as a limitation per PRISMA-ScR item 5. PROSPERO does not accept scoping review protocols; to mitigate this, the pre-specified internal protocol has subsequently been deposited in Zenodo with a permanent identifier (https://doi.org/10.5281/zenodo.19641610), and the search, eligibility criteria, and charting categories were not altered after the search was initiated. No formal risk-of-bias assessment was performed, consistent with scoping review methodology [14–16]. We could not determine the prevalence of resistant organisms in the four definitive RCTs because this information was not reported. Only English-language publications were included.

## Conclusions

Current RCT and meta-analytic evidence consistently supports noninferiority of 7-day therapy for uncomplicated BSI caused by susceptible organisms, a conclusion reinforced by six independent meta-analyses and a convergent body of observational data. For resistant BSI, the preliminary evidence is reassuring but not definitive: the few available studies show no clear signal of harm with shorter courses, but none had the statistical power to establish noninferiority. The absence of such evidence should not be equated with evidence of equivalence. Clinicians treating resistant BSI should weigh pathogen characteristics, source control, clinical response, host factors, and antibiotic bioavailability on a case-by-case basis. Resistance-stratified duration trials—most feasibly through platform trial architectures such as BALANCE + and pathogen-specific studies such as SHORTEN-2—are the priority the field now requires [32, 34].

## Data Availability

All data used in this scoping review are from published studies and are available in the public domain. No new data were generated.

## Declarations

### Transparency declaration

The lead author affirms that this manuscript is an honest, accurate, and transparent account of the study being reported; that no important aspects of the study have been omitted; and that any discrepancies from the study as planned have been explained.
